# Molecular interaction of 1-aminocyclopropane-1-carboxylate deaminase (ACCD)-producing endophytic *Streptomyces* sp. GMKU 336 towards salt-stress resistance of *Oryza sativa* L. cv. KDML105

**DOI:** 10.1038/s41598-018-19799-9

**Published:** 2018-01-31

**Authors:** Ratchaniwan Jaemsaeng, Chatchawan Jantasuriyarat, Arinthip Thamchaipenet

**Affiliations:** 1 Department of Genetics, Faculty of Science, Kasetsart University, Bangkok, 10900 Thailand; 2 Center for Advanced Studies in Tropical Natural Resources, National Research University-Kasetsart University (CASTNAR, NRU-KU), Bangkok, 10900 Thailand

## Abstract

1-aminocyclopropane-1-carboxylate deaminase (ACCD)-producing endophytic *Streptomyces* sp. GMKU 336 and its ACCD-deficient mutant were inoculated into Thai jasmine rice Khao Dok Mali 105 cultivar (*Oryza sativa* L. cv. KDML105) under salt stress (150 mM NaCl) conditions. The results clearly indicated that *Streptomyces* sp. GMKU 336 significantly increased plant growth, chlorophyll, proline, K^+^, Ca^+^, and water contents; but decreased ethylene, reactive oxygen species (ROS), Na^+^, and Na^+^/K^+^ ratio when compared to plants not inoculated and those inoculated with the ACCD-deficient mutant. Expression profiles of stress responsive genes in rice in association with strain GMKU 336 were correlated to plant physiological characteristics. Genes involved in the ethylene pathway, *ACO1* and *EREBP1*, were significantly down-regulated; while *acdS* encoding ACCD in *Streptomyces* sp. GMKU 336 was up-regulated *in vivo*. Furthermore, genes involved in osmotic balance (*BADH1*), Na^+^ transporters (*NHX1* and *SOS1*), calmodulin (*Cam1-1*), and antioxidant enzymes (*CuZn-SOD1* and *CATb*) were up-regulated; whereas, a gene implicated in a signaling cascade, *MAPK5*, was down-regulated. This work demonstrates the first time that ACCD-producing *Streptomyces* sp. GMKU 336 enhances growth of rice and increases salt tolerance by reduction of ethylene via the action of ACCD and further assists plants to scavenge ROS, balance ion content and osmotic pressure.

## Introduction

Salinity is one of the major environment stress factors that reduces plant cell division, growth and productivity. Recently, plant growth-promoting (PGP) bacteria have been identified that enhance tolerance to salinity by plants, particularly bacteria associated with the plants^1^. Endophytic actinomycetes are of special interest because they not only produce various bioactive secondary metabolites to protect plants from infectious diseases^2^, but they also show ability to enhance plant growth by carrying several PGP traits including production of siderophores to capture iron, production of plant hormones such as auxins and cytokinins, solubilization of phosphate and other minerals to supply nutrients^3,4^. Moreover, they facilitate plant growth under stress caused by drought, heavy metals, flooding and high salt by reducing stress associated with ethylene via production of 1-aminocyclopropane-1-carboxylate (ACC) deaminase^5,6^.

Ethylene has long been recognized as a hormone that controls plant responses to growth limiting conditions. Under stress conditions, the ethylene level is increased via the ethylene pathway that transforms the precursor ACC into the final product, ethylene^1^. A diverse group of endophytic PGP bacteria is able to reduce plant ethylene levels by the action of ACC deaminase (ACCD). ACCD (encoded by the *acdS* gene) converts ACC in plants to ammonia and α-ketobutyrate, which the bacteria consume as nitrogen sources^7^. Therefore, ACCD-producing bacteria stimulate plant ACC efflux and subsequently decrease ACC concentration and ethylene production^1^. The consequence of this interaction is an increase in root/shoot elongation and protection of the plant from the inhibitory effects of ethylene^8^. Thus, plants associated with endophytic ACCD-producing bacteria become more resistant to stress.

So far, the role and interaction of ACCD-producing endophytic actinomycetes to promote plant growth under salt stress has been less studied. This research therefore focused on ACCD-producing endophytic *Streptomyces* sp. GMKU 336 and its ability to enhance the growth of rice under salt stress conditions. An ACCD-deficient mutant of *Streptomyces* sp. GMKU 336 was constructed and compared with the effect of growth promotion of rice with the wild type under salt stress *in situ*. This work demonstrated that strain GMKU 336 increases salt tolerance of salt-sensitive Thai jasmine rice Khao Dok Mali 105 cultivar (KDML105). Expression profiles of stress responsive genes of rice associated with strain GMKU 336 are demonstrated and the impact of the interaction is discussed. Understanding of such interaction will lead to sustainably utilize ACCD-producing endophytic actinomycetes to enhance growth and salt tolerance in rice growing in saline soil.

## Results

### Characterization of plant growth promoting traits of *Streptomyces* sp. GMKU 336

As part of a program to discover PGP endophytic actinomycetes from medicinal plants, *Streptomyces* sp. GMKU 336 was recovered from the roots of *Clerodendrum serratum* (L.) Moon^9^. The strain is most closely related to *Streptomyces hydrogenans* NBRC 13475 ^T^, with 99.86% identity based on 16S rDNA gene sequence analysis (GenBank accession number KR870352). Screening of plant growth promoting (PGP) traits of strain GMKU 336 revealed characteristics of phosphate solubilization, siderophore production, 1-aminocyclopropane-1-carboxylate deaminase (ACCD) activity but no indole-3-acetic acid (IAA) production (data not shown). Strain GMKU 336 shows moderate halophilic type as it tolerates NaCl up to 6% (w/v).

### Construction of ACCD-deficient mutant

*Streptomyces* sp. GMKU 336 displayed ACCD activity at 2.85 ± 0.15 μmol α-ketobutyrate mg protein^−1^ h^−1^. Furthermore, the expression profile of the ACCD gene (*acdS*) by semi-quantitative RT-PCR revealed high expression when bacteria were consuming ACC as a sole nitrogen source (data not shown). An ACCD-deficient mutant of strain GMKU 336 was then constructed by insertional inactivation of *acdS* (GenBank accession number KT000002). The mutant constructed showed no ACCD activity and the disruption was verified by PCR analysis (Supplementary Fig. S1). The mutant was stable up to five generation of growth without thiostrepton selection. The mutant was reverted to wild type by further selection without the antibiotic up to ten generations. The revertant showed the same ACCD activity and all other properties as wild type (data not shown).

### Effect of ACCD-producing *Streptomyces* sp. GMKU 336 inoculated rice plants under salt stress

*Streptomyces* sp. GMKU 336 and its ACCD-deficient mutant were inoculated into KDML105. The growth parameters were observed after 7 days of treatment with 150 mM NaCl under hydroponic conditions and compared with non-salt treatment (Supplementary Fig. S2 and Supplementary Table S1). Re-isolation of wild type and mutant from both salt and non-salt treatments was about 10^4^ CFU g root fresh weight^−1^ (Supplementary Table S1). Both strains were confirmed by 16S rRNA gene sequencing and PCR analysis (data not shown). In addition, un-inoculated plants were shown not to harbor *Streptomyces* sp. GMKU 336 as well as other endophytic actinomycetes. The results indicated that the surface sterilized protocol of rice seeds was successful and the hydroponic condition used in this study are free from contamination.

**Figure 1:**
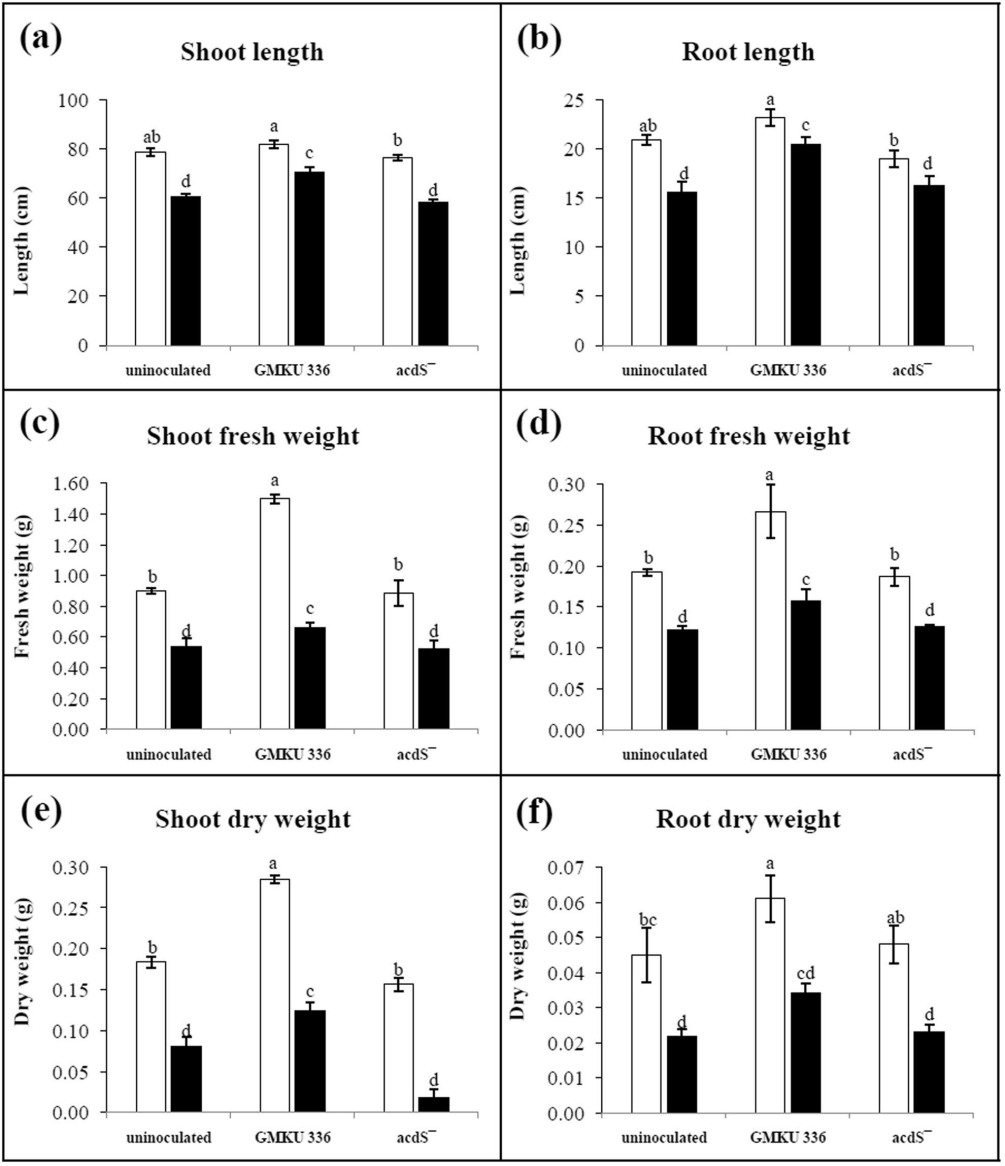
Effect of ACCD-producing *Streptomyces* sp. GMKU 336 on growth of *Oryza sativa* L. cv. KDML105 after 7 days of salt stress under hydroponic conditions. (**a**) Shoot length; (**b**) root length, (**c**) shoot fresh weight; (**d**) root fresh weight; (**e**) shoot dry weight; (**f**) root dry weight. Values are mean of three replicates ± standard error of mean. Different letters indicated statistical differences between treatments (Duncan’s test, P < 0.05). Uninoculated, plants without bacteria inoculation; GMKU 336, plants inoculated with *Streptomyces* sp. GMKU 336; acdS^−^, plants inoculated with ACCD-deficient mutant; white bar, non-salt treatment; black bar, salt treatment (150 mM NaCl).

Under non-salt conditions, ACCD-producing *Streptomyces* sp. GMKU 336 slightly enhanced plant elongation compared to un-inoculated controls (Fig. 1a and b), but significantly increased plant biomass (1.2–1.6 fold) including shoot/root fresh and dry weights (Fig. 1c–f). Rice inoculated with the ACCD-deficient mutant showed similar plant growth parameters to those of un-inoculated controls (Fig. 1). Under salt-stress conditions, all growth parameters were significantly reduced when compared to the non-salt treatments (Fig. 1). Therefore, strain GMKU 336 was able to promote growth of KDML105 with or without salt treatment.

A symptom of salt toxicity in the KDML105 was evaluated using the standard scoring protocol^10^. Under salt treatment, complete cessation of growth was scored as ‘susceptible’ in un-inoculated plants and those inoculated with the ACCD-deficient mutant, whereas nearly normal growth was scored as ‘tolerant’ in plants inoculated with strain GMKU 336 (Supplementary Fig. S2 and Supplementary Table S1).

### Effect of ACCD-producing *Streptomyces* sp. GMKU 336 on plant physiology

**Figure 2:**
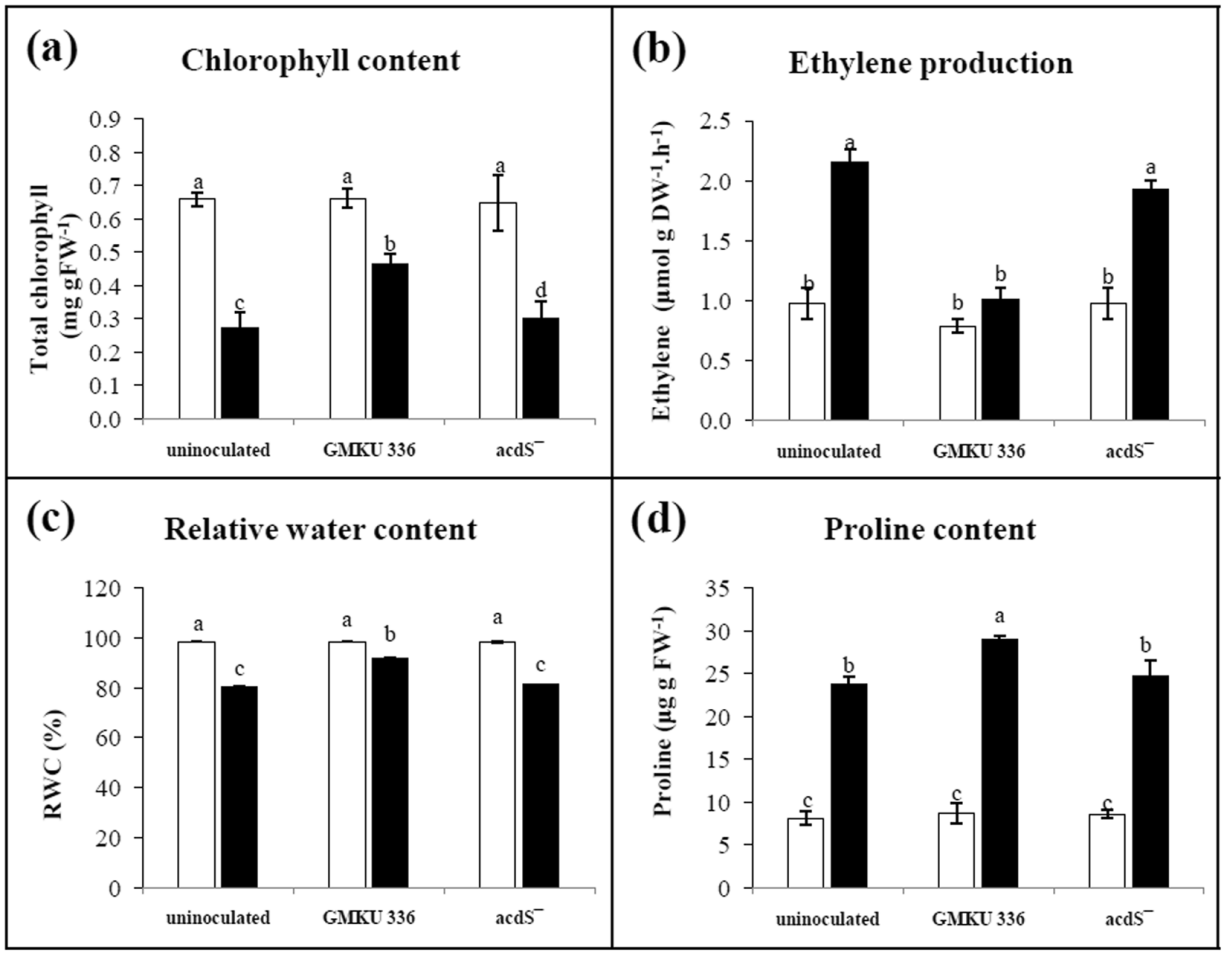
Effect of ACCD-producing *Streptomyces* sp. GMKU 336 on plant physiology of *Oryza sativa* L. cv. KDML105. (**a**) Chlorophyll content; (**b**) ethylene production; (**c**) relative water content; (**d**) proline content. Values are mean of three replicates ± standard error of mean. Different letters indicated statistically differences between treatments (Duncan’s test, P < 0.05). Uninoculated, plants without bacteria inoculation; GMKU 336, plants inoculated with *Streptomyces* sp. GMKU 336; acdS^−^, plants inoculated with ACCD-deficient mutant; white bar, non-salt treatment; black bar, salt treatment (150 mM NaCl).

Indices of plant physiology including chlorophyll content, ethylene production, relative water content, and proline content revealed constant level of each parameters of all treatments of non-salt stressed plants (Fig. 2). Under salt-stress conditions, a significant decrease in chlorophyll content of rice was observed in all treatments, compared to those grown in non-salt conditions. However, the KDML105 inoculated with *Streptomyces* sp. GMKU 336 had a significantly higher (1.7-fold) chlorophyll content compared to that of an un-inoculated control (Fig. 2a, Supplementary Table S1). Significant induction of ethylene production (2-fold) was observed in un-inoculated plants and those inoculated with the ACCD-deficient mutant, compared to those of non-salt treatment (Fig. 2b, Supplementary Table S1). Remarkably and in contrast to that of non-salt treatment, KDML105 inoculated with strain GMKU 336 had no increment in ethylene level (Fig. 2b). The water content of all treatments was drastically reduced after exposure to salt stress. Nevertheless, plants inoculated with strain GMKU 336 accumulated water significantly more than the un-inoculated control (Fig. 2c). Furthermore, proline content was increased in all salt-stressed plants, but those inoculated with strain GMKU 336 had significantly higher proline content than un-inoculated controls (Fig. 2d). The results suggested that strain GMKU 336 has a positive effect on the physiology of rice to tolerate salinity.

### Effect of ACCD-producing *Streptomyces* sp. GMKU 336 on plant ion content

**Figure 3:**
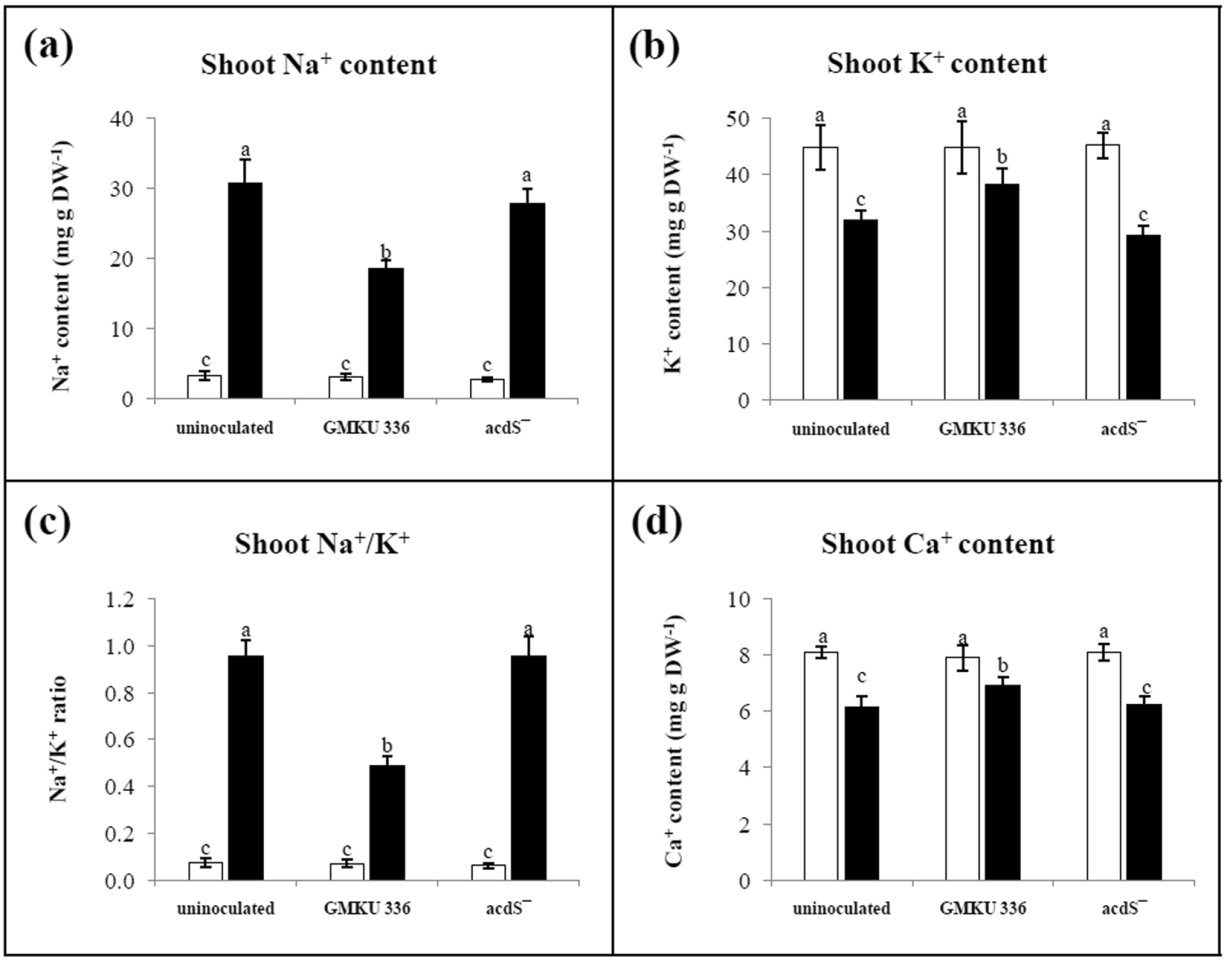
Effect of ACCD-producing *Streptomyces* sp. GMKU 336 on ion content of shoots of *Oryza sativa* L. cv. KDML105. (**a**) Na^+^ content; (**b**) K^+^ content; (**c**) Na^+^/K^+^ ratio; (**d**) Ca^2+^ content. Values are mean of three replicates ± standard error of mean. Different letters indicated statistically significant differences between treatments (Duncan’s test, P < 0.05). Uninoculated, plants without bacteria inoculation; GMKU 336, plants inoculated with *Streptomyces* sp. GMKU 336; acdS^−^, plants inoculated with ACCD-deficient mutant; white bar, non-salt treatment; black bar, salt treatment (150 mM NaCl).

Under normal conditions, the ion content of KDML105, specifically Na^+^, K^+^, and Ca^2+^, was at the same level in all treatments (Fig. 3). Under salt-stress conditions, the Na^+^ content of all plant treatments was significantly increased (Fig. 3a). Un-inoculated plants and those inoculated with the ACCD-deficient mutant had a Na^+^ content nearly 10-fold higher than plants with the corresponding non-salt treatment, whereas plants inoculated with *Streptomyces* sp. GMKU 336 had a Na^+^ content that was only 6-fold higher than the non-salt control (Fig. 3a, Supplementary Table S1). By contrast, the K^+^ content of all plant treatments was significantly decreased under salt-stress conditions (Fig. 3b). However, plants inoculated with strain GMKU 336 had a smaller decrease in K^+^ content compared to un-inoculated plants and those inoculated with the ACCD-deficient mutant (Fig. 3b). Taken together, there was a 13-fold increment in Na^+^/K^+^ ratio for un-inoculated plants and those inoculated with the ACCD-deficient mutant when compared to the non-salt treatment. On the other hand, the increment in Na^+^/K^+^ ratio was only 6-fold in plants inoculated with strain GMKU 336 (Fig. 3c, Supplementary Table S1). Salt stress also caused a decrease in Ca^2+^ content in all plant treatments (Fig. 3d). However, plants inoculated with strain GMKU 336 had significantly less reduction in Ca^2+^ content than un-inoculated plants or those inoculated with the ACCD-deficient mutant (Fig. 3d). The results implied that strain GMKU 336 helps maintain ion balance and, thus, increases salt tolerance in KDML105.

### Effect of ACCD-producing *Streptomyces* sp. GMKU 336 on reactive oxygen species (ROS)

**Figure 4:**
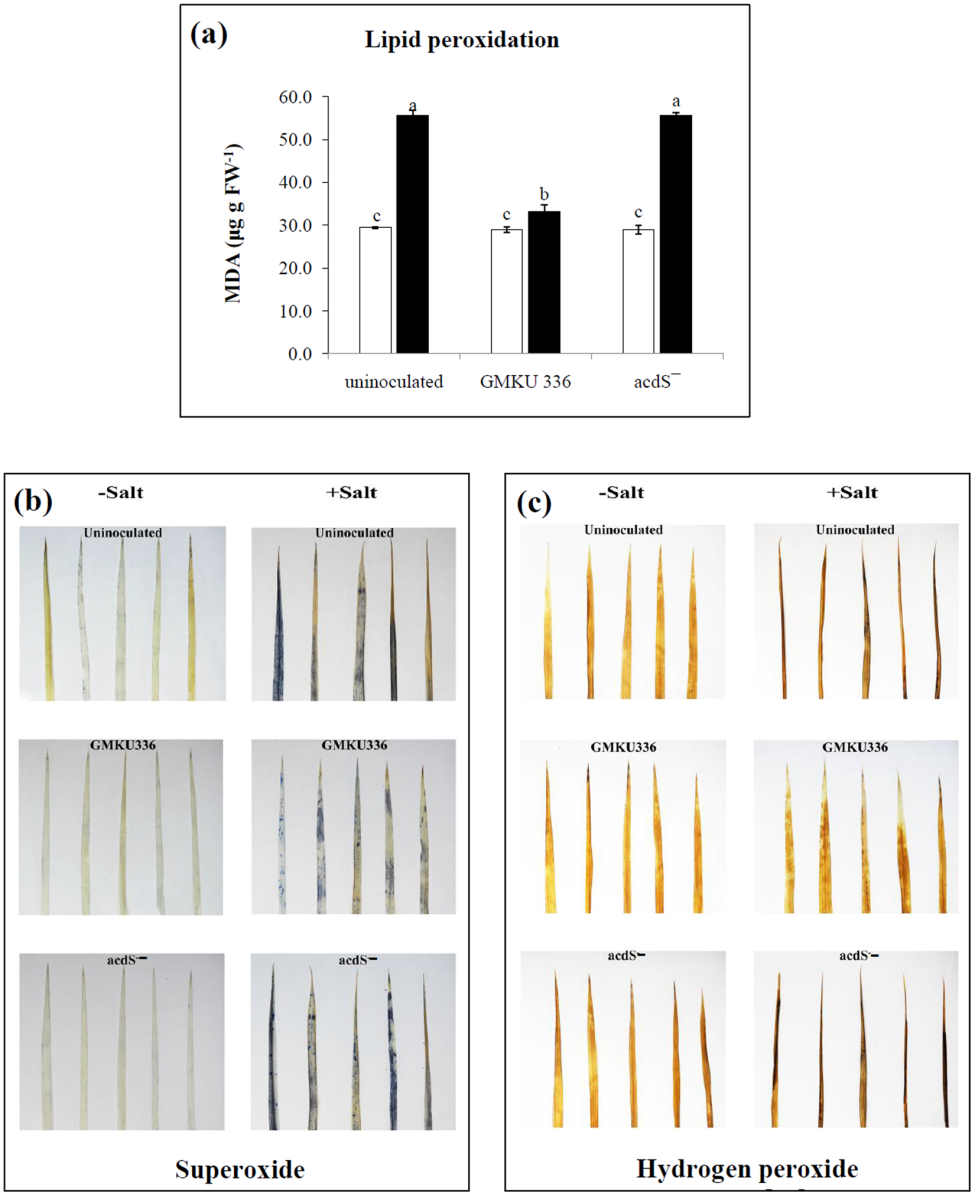
Effect of ACCD-producing *Streptomyces* sp. GMKU 336 in reactive oxygen species (ROS) in *Oryza sativa* L. cv. KDML105. (**a**) lipid peroxidation; (**b**) superoxide by NBT staining; (**c**) hydrogen peroxide by DAB staining. Values are mean of three replicates ± standard error of mean. Different letters indicated statistical differences between treatments (Duncan’s test, P < 0.05). Uninoculated, plants without bacteria inoculation; GMKU 336, plants inoculated with *Streptomyces* sp. GMKU 336; acdS^−^, plants inoculated with ACCD-deficient mutant; white bar, non-salt treatment; black bar, salt treatment (150 mM NaCl).

A significant increase of lipid peroxidation determined by estimating production of malondialdehyde (MDA) content was observed in all plant treatments exposed to salt (Fig. 4a). Un-inoculated plants and those inoculated with the ACCD-deficient mutant accumulated MDA nearly 2-fold higher than the non-salt treatments. By contrast, plants inoculated with *Streptomyces* sp. GMKU 336 had a MDA content less than half of both treatments (Fig. 4a, Supplementary Table S1). ROS were detected in leaves by staining with nitrobluetrazolium (NBT) (Fig. 4b) and 3,3′-diaminobenzidine (DAB) (Fig. 4c), which indicate the presence of superoxide and hydrogen peroxide, respectively. In the presence of salt, leaves had higher staining indicative of both ROS species; however, plants inoculated with strain GMKU 336 showed less staining than the other treatments (Fig. 4b and c).The results suggested that strain GMKU 336 reduces ROS in salt-stressed rice.

### Effect of ACCD-producing *Streptomyces* sp. GMKU 336 on expression profile of genes involved in the ethylene pathway

**Figure 5:**
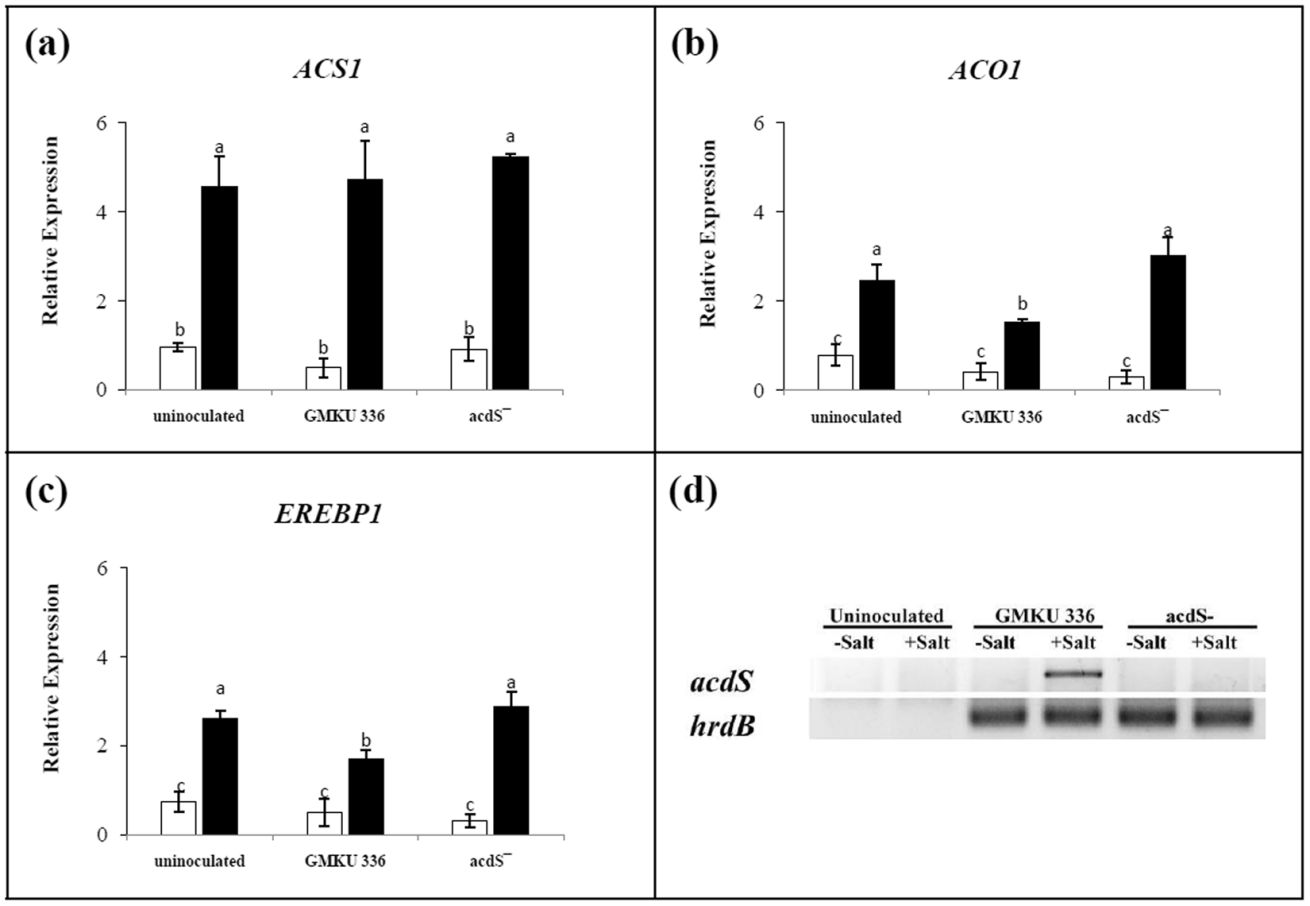
Transcriptional analysis of genes involved in ethylene production in *Oryza sativa* L. cv. KDML105 associated with *Streptomyces* sp. GMKU 336. (**a**) ACC synthase (*ACS1*); (**b**) ACC oxidase (*ACO1*); (**c**) ethylene responsive element binding proteins (*EREBP1*); (**d**) ACCD (*acdS*) of *Streptomyces* sp. GMKU 336. The two gels cropped from different gels (Supplementary Fig. S3). The histogram represents mean of the expression ratio, relative to the actin gene (*act1*). Values are mean of three replicates ± standard error of mean. Different letters indicated statistically-significant differences between treatments (Duncan’s test, P < 0.05). Uninoculated, plants without bacteria inoculation; GMKU 336, plants inoculated with *Streptomyces* sp. GMKU 336; acdS^−^, plants inoculated with ACCD-deficient mutant; *hrdB*, RNA polymerase principal sigma factor gene of *Streptomyces* sp. GMKU 336; white bar/−Salt, non-salt treatment; black bar/+Salt, salt treatment (150 mM NaCl).

As KDML105 inoculated with *Streptomyces* sp. GMKU 336 maintained the same level of ethylene either with or without salt treatment similar to those un-inoculated plants and those inoculated with the ACCD-deficient mutant under non-salt condition (Fig. 2b), gene expression patterns of ACC synthase (*ACS1*), ACC oxidase (*ACO1*) and the ethylene responsive element binding protein (*EREBP1*) were investigated by real-time PCR. All three genes were expressed at the same basal level in all plant treatments under non-salt conditions (Fig. 5a–c). The expression level of *ACS1* was about 5-fold up-regulated in all salt-stressed plants compared to the corresponding non-salt controls (Fig. 5a). The expression profiles of *ACO1* and *EREBP1* in un-inoculated plants and those inoculated with the ACCD-deficient mutant were about 3–4 fold higher than the non-salt treatments, whereas plants inoculated with strain GMKU 336 had 1-fold lower expression (Fig. 5b, Supplementary Table S2). Moreover, expression of the *acdS* gene encoding ACCD in strain GMKU 336 was only detected *in vivo* within salt-stressed rice (Fig. 5d). The results indicated that strain GMKU 336 reduces ethylene content through the action of ACCD and consequently down-regulated *ACO1* and *EREBP1* genes in rice.

### Effect of ACCD-producing *Streptomyces* sp. GMKU 336 on expression of salt-stress responsive genes

**Figure 6:**
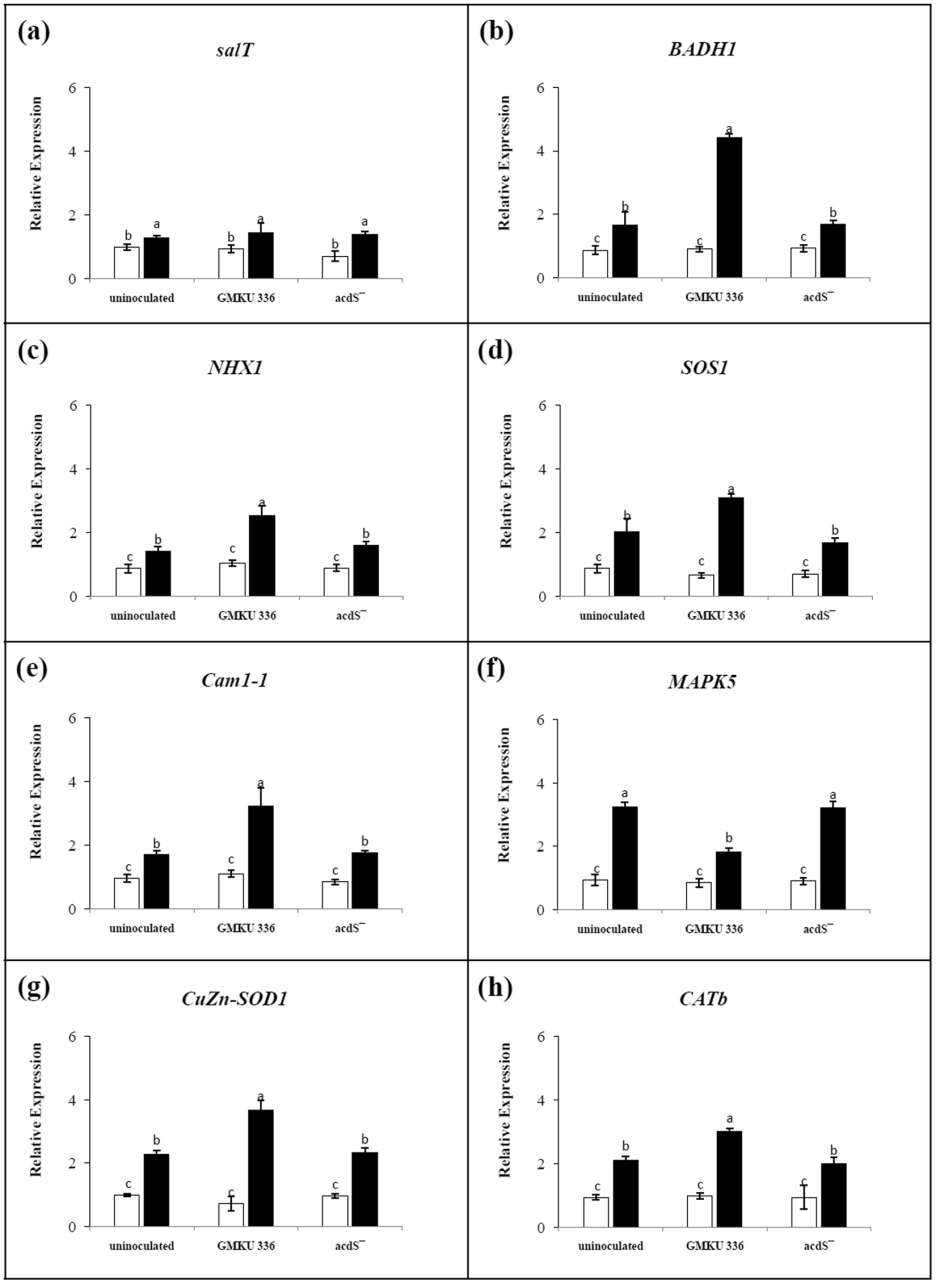
Transcriptional analysis of genes involved in salt stress response in *Oryza sativa* L. cv. KDML105 associated with *Streptomyces* sp. GMKU 336. (**a**) Salt stress responsive protein (*salT*); (**b**) betaine aldehyde dehydrogenase (*BADH1*); (**c**) Na^+^/H^+^ antiporter (*NHX1*); (**d**) salt overlay sensitive 1 protein (*SOS1*); (**e**) calmodulin (*Cam1-1*); (**f**) mitogen activated protein kinase (*MAPK5*); (**g**) superoxide dismutase (*CuZn-SOD1*); (**h**) catalase (*CATb*). The histogram represents mean of expression ratio, relative to the actin gene (*act1*). Values are mean of three replicates ± standard error of mean. Different letters indicated statistically-significant differences between treatments (Duncan’s test, P < 0.05). Uninoculated, plants without bacteria inoculation; GMKU 336, plants inoculated with *Streptomyces* sp. GMKU 336; acdS^−^, plants inoculated with ACCD-deficient mutant; white bar, non-salt treatment; black bar, salt treatment (150 mM NaCl).

Eight candidate genes of rice encoded proteins involved in the salt stress response were transcriptionally analyzed in un-inoculated plants and those inoculated with wild type or mutant bacteria, under salt and non-salt conditions. The genes were: *salT* (salt stress responsive protein)^11^, *BADH1* (betaine aldehyde dehydrogenase)^12^, *NHX1* (Na^+^/H^+^ antiporter)^13^, *SOS1* (salt overlay sensitive 1 protein)^13^, *Cam1-1* (calmodulin)^14^, *MAPK5* (mitogen activated protein kinase 5)^12^, *CuZn-SOD1* (superoxide dismutase)^15^, and *CATb* (catalase)^12^. Expression profiles of all genes were observed at similar basal level in plants grown under non-salt conditions (Fig. 6). When KDML105 were exposed to salt, the salt-induced positive control gene, *salT*, was up-regulated to a similar level in all treatments (Fig. 6a). The expression profile of *BADH1*, involved in competition of solute production, was up-regulated in all salt-stressed plants. However and significantly, it was 4-fold higher in plants inoculated with *Streptomyces* sp. GMKU 336 compared to un-inoculated plants and those inoculated with the ACCD-deficient mutant (Fig. 6b, Supplementary Table S2).

Genes involved in Na^+^ transport, specifically a member of vacuole Na^+^/H^+^ transporters (*NHX1*) and salt overlay sensitive 1 protein (*SOS1*), were significantly (1.6–2.3 fold) up-regulated in salt-stressed un-inoculated plants and those inoculated with the ACCD-deficient mutant, when compared to the non-salt controls. Rice inoculated with strain GMKU 336 had expression levels that were 3–4 fold higher than control (Fig. 6c and d, Supplementary Table S2). Up-regulation of the *Cam1-1* gene, a Ca^+^ sensor involved in plant signaling by calmodulin, was 1.8-fold higher than the non-salt treatment in salt-stressed un-inoculated plants and those inoculated with the ACCD-deficient mutant. However, plants inoculated with strain GMKU 336 had expression levels that were 1-fold higher than both treatments (Fig. 6e, Supplementary Table S2). High expression of the *MAPK5* gene, encoding a kinase protein, was detected in un-inoculated plants and those inoculated with the ACCD-deficient mutant when compared to those of non-salt treatments. By contrast, plants inoculated with strain GMKU 336 had nearly 2-fold expression when compared to its control (Fig. 6f, Supplementary Table S2).

Further analysis of gene expression encoding antioxidant enzymes, superoxide dismutase (*CuZn-SOD1*) and catalase (*CATb*), revealed 2.1–2.3 fold up-regulation in salt-stressed un-inoculated plants and those inoculated with the ACCD-deficient mutant when compared to the non-salt treatments. Significantly, expression levels of plants inoculated with strain GMKU 336 were 3.2–3.7 times higher than the non-salt controls (Fig. 6g and h, Supplementary Table S2). All of these results indicate that strain GMKU 336 has a positive influence to salt stress response gene expression in KDML105 by up-regulation of *BADH1*, *NHX1*, *SOS1*, *Cam1-1*, *CuZn-SOD1*, and *CATb*, and down-regulation of *MAPK5*, and increases salt tolerant in rice as a consequence.

## Discussion

Recently, actinomycetes have been reported to promote plant growth as well as alleviate various abiotic stresses including salinity and osmotic stress via the action of 1-aminocyclopropane-1-carboxylate deaminase (ACCD)^5,6^. However, the role of ACCD-producing endophytic actinomycetes in promoting plant growth under stress conditions has not yet been investigated systematically. *Streptomyces* sp. GMKU 336 was used in this study as it displayed highest ACCD activity amongst other strains in the screening program^9^ and showed halophilic property which is suitable to investigate the *in vivo* molecular interactions of this strain in partnership with the salt-sensitive Thai jasmine rice Khao Dok Mali 105 cultivar (KDML105) under salt-stress conditions. In addition, strain GMKU 336 revealed endophytic ability in rice although it was isolated from medicinal plant. The result was in agreement with previous report that endophytic *Streptomyces* isolated from one plant species could mutually resided in the other different plant species by *in vitro* inoculation^3^. In this work, an ACCD-deficient mutant of strain GMKU 336 was constructed by insertional inactivation of the *acdS* gene to define definitively the role of ACCD on plant growth and salt tolerance.

Under non-salt conditions, KDML105 inoculated with ACCD-producing *Streptomyces* sp. GMKU 336 had significantly enhanced shoot and root biomass but not obviously extended shoot and root lengths. This might be due to the lack of IAA production of this strain that would not encourage the elongation of plants. However, the results for plant growth were consistent with previous work showing that ACCD-producing *Streptomyces* have an ability to enhance growth of tomato^5^, *Arabidopsis*^16^, halophytic *Limonium sinense*^17^ and sugarcane^4^. The growth effect has also been found in other bacteria such as ACCD-producing *Pseudomonas*^18^, *Enterobacter*^19^, and *Bacillus*^20^ that enhanced growth of canola.

Addition of salt had a negative effect on plant growth parameters in all plant treatments. However, KDML105 inoculated with *Streptomyces* sp. GMKU 336 maintained high shoot and root elongation when compared to un-inoculated plants and those inoculated with the ACCD-deficient mutant. The results were in agreement with previous work that an ACCD-producing *Streptomyces* increased shoot and root growth of halophytic *Ligustrum sinense* under salt-stress treatment^6^. ACCD-producing *Bacillus* significantly increased seed germination and promoted growth of rice KDML105^21^ and indica rice^12^. Likewise, an ACCD-producing *Pseudomonas* increased yield and enhanced salt resistance in various plants including canola^22^ and tomato^23^ which was not observed with its *acdS* mutant strain. Since KDML105 is a salt-sensitive cultivar^24^, symptoms of salt toxicity were observed in un-inoculated plants and those inoculated with the ACCD-deficient mutant. By contrast, inoculation of KMDL105 with strain GMKU 336 resulted in the rice exhibiting better resistance to salinity stress.

Salt stress reduced chlorophyll content in all KDML105 treatments. However, plants inoculated with *Streptomyces* sp. GMKU 336 had less reduction of chlorophyll content. The result was similar to that with PGP *Azospirillum*^25^ and ACCD-producing *Bacillus*^12^ which significantly increased chlorophyll content in respective maize and rice grown in high salt conditions. Furthermore, salt stress significantly accelerated ethylene synthesis in all rice treatments. By contrast, rice inoculated with strain GMKU 336 maintained ethylene content at the same level as that of non-salt treatment. The result is supported by other reports that ACCD-producing *Pseudomonas* suppressed ethylene synthesis and decelerated chlorophyll decay in wheat under salt-stress conditions^26^. Besides, lower amount of ACC, a precursor of ethylene, was observed in canola inoculated with ACCD-producing *Pseudomonas*^18^ and *Enterobacter*^19^. It was also reported that *Streptomyces* enhanced plant growth by lowering the plant ACC and ethylene levels in tomato^5^.

Salt stress induced high activities of ACC synthase and ACC oxidase and subsequently produced high levels of ethylene^1^. In this work, expression profiles of rice genes involved in the ethylene pathway including ACC synthase (*ACS1*) and ACC oxidase (*ACO1*) were up-regulated in all plants treated with salt. Here, we report the remarkable reduction of ethylene in KDML105 inoculated with *Streptomyces* sp. GMKU 336, which is correlated with low expression of the *ACO1* gene. Expression of the *acdS* gene encoding ACCD of strain GMKU 336 was only observed *in vivo* with salt-treated rice. The results indicated that lower expression of the *ACO1* gene of salt-treated rice inoculated with strain GMKU 336 was due to the expression of *acdS* gene of the bacteria which converted the ACC to ammonia and α-ketobutyrate and subsequently reduced the level of the stress molecule, ethylene. The results were in agreement with the expression of *acdS* of *Mesorhizobium* spp.^27^ and *Sinorhizobium* sp. BL3^28^ in nodules of chickpea and mungbean, respectively under salinity condition. However, the expression was not detected in mungbean inoculated with an *acdS*-deficient mutant of strain BL3^28^. The gene encoding an ethylene responsive element binding protein (*EREBP1*) was up-regulated in all plants treated with salt. EREBP is a member of the ethylene-response factor (ERF) family^29^, which plays an important role in abiotic stress response^30^. Up-regulation of *EREBP1* was observed in tobacco during drought and salt stress^31^. Here, we report that KDML105 inoculated with *Streptomyces* sp. GMKU 336 expressed *EREBP1* at significantly lower levels. Canola inoculated with ACCD-producing *Pseudomonas putida* UW4 had reduced expression of *ERF*, while plants inoculated with an ACCD-deficient mutant had increased the expression^32^.

Under salt-stress conditions, plants adapt by producing competition solutes such as proline and glycine betaine that help to stabilize proteins and cell structures, osmotic balance, scavenge reactive oxygen species (ROS)^33^, and increases chlorophyll content^34^. Here we report that the water and proline contents of all salt-treated rice were significantly increased, but highly accumulated in plants inoculated with *Streptomyces* sp. GMKU 336. The results correlated with previous reports that PGP *Dietzia*^35^ and ACCD-producing *Bacillus*^12^ improved salt tolerance in respective wheat and rice by enhancement of proline content. Furthermore, the transcription profile of the betaine aldehyde dehydrogenase gene (*BADH1*) that converts choline to glycine betaine was up-regulated in all salt-stressed rice and expressed at the highest level in plants associated with strain GMKU 336. The result was in agreement with ACCD-producing *Bacillus* that maintained osmotic adjustment in rice under salinity condition by accumulation of glycine betaine and up-regulation of the *BADH1* gene^12^.

It is generally known that the maintenance of low cytosolic Na^+^ concentrations and Na^+^/K^+^ homeostasis are important for tolerance to salinity. ACC could promote the production of ethylene and improve the response to salinity-induced injury by homeostasis of Na^+^/K^+^ ^12^. High accumulation of Na^+^ inside the cells inhibits K^+^ uptake and results in an increase in Na^+^/K^+^ ratio that is inversely related to the level of salt tolerance^36^. Here we report that decreased Na^+^ content and increased K^+^ resulted in a reduction of the Na^+^/K^+^ ratio in salt-stressed rice inoculated with *Streptomyces* sp. GMKU 336. It was reported that PGPB may regulate the uptake of Na^+^/K^+^ and maintain a nutritional balance in plants^37^. The results were in agreement with previous reports that cotton inoculated with ACCD-producing *Klebsiella* showed high K^+^ concentrations that resulted in enhancement of salt tolerance^38^.

Increases in the uptake of Na^+^ in shoot vacuoles could enhance salt tolerance in plants. Therefore, the most direct way to manage excess cytoplasmic Na^+^, which is toxic to plant cells, is to pump the excess Na^+^ to a vacuole catalyzed by a Na^+^/H^+^ antiporter^13^. We observed up-regulation of the Na^+^/H^+^ antiporter gene (*NHX1*) in all salt-stressed rice, which was significantly highest in plants inoculated with *Streptomyces* sp. GMKU 336. Similarly, high expression level of *NHX* was observed in wheat inoculated with PGP *Dietzia* which correlated with enhancement of salt tolerance^35^. Na^+^ efflux is one of the mechanisms that maintains the level of Na^+^ in the cytoplasm. Salt overlay sensitive 1 (SOS1) is the only Na^+^ efflux protein located at the plant plasma membrane^13^. We report here that expression of the *SOS1* gene was increased in all salt stressed rice and significantly highest in plants inoculated with strain GMKU 336. The results were in agreement with other reports of overexpression of the *SOS1* gene in salt tolerant rice^39^ and *Arabidopsis*^40^. The results indicated that excess Na^+^ was reduced by the up-regulation of *NHX1* and *SOS1* genes which was an effect of strain GMKU 336 to enhance growth and salt tolerance of rice.

The calcium signaling network is one of the signal cascades involved in transient changes in cytosolic Ca^2+^ concentration, which was reported to be a key messenger in the salt stress response^41^. A decrease in Ca^2+^ content under stress condition was previously reported in KDML105^24^ and other rice salt-sensitive lines^42^. Here we report a decrease in Ca^2+^ content as a result of salt stress in all salt treated rice. However, plants inoculated with *Streptomyces* sp. GMKU 336 maintained a significantly higher Ca^2+^ content. Increase of Ca^2+^ content was also observed in eggplant^43^ and cotton^44^ inoculated with PGP *Pseudomona*s. Furthermore, the expression profile of *Cam 1-1*, involved in calmodulin, was significantly up-regulated in all salt-stressed rice, but highest in plants inoculated with strain GMKU 336. The binding of Ca^2+^ to the calmodulin complex is able to regulate a variety of cellular processes implicated in salt and other stresses^14^, therefore, *Cam1-1* gene is a significant player in the Ca^2+^ signal transduction network. The responses of high Ca^2+^ content and *Cam1-1* gene expression on rice KDML105 to salt stress suggested that strain GMKU 336 plays a positive role to induce calmodulin and Ca^2+^ content to help rice tolerate salinity.

The response of plant cells to salt stress is controlled by multiple mechanisms linked to stress and other developmental responses. Ethylene was additionally reported to mediate crosstalk between mitogen activated protein kinase (MAPK) signaling pathways^45^. Plant MAPK cascades are thought to play a key role in biotic and abiotic stress responses, hormone response, cell division and development in rice^46^. We report here that the expression profile of *MAPK5* was significantly up-regulated in all salt-stressed rice, but had lower expression in plants inoculated with *Streptomyces* sp. GMKU 336. Similarly, a low expression level of *MAPK5* was observed in rice inoculated with *Bacillus amyloliquefaciens* NBRISN13 that increased salt tolerance of rice in soil^12^. However, *MAPK* gene expression profiles of rice^47^, pea^48^, and *Arabidopsis*^49^, were up-regulated during ethylene induction. The results suggested that lower ethylene production in rice inoculated with strain GMKU 336 under salt-stress treatment might reflect the low expression level of the *MAPK5* gene.

In rice, salinity triggers MAPK cascades to stabilize ACC synthase activity that enhances ethylene production and ethylene signaling, which then promotes ROS accumulation leading to lipid peroxidation (high accumulation of MDA content) and growth inhibition^50^. In this experiment, salinity significantly induced high accumulation of MDA content and ROS including superoxide and hydrogen peroxide in all salt-stressed rice. Remarkably, *Streptomyces* sp. GMKU 336 reduced ROS leading to a reduction in lipid peroxidation in rice. Earlier studies suggested that induction of ROS scavenging anti-oxidative enzymes such as superoxide dismutase (SOD) and catalase (CAT) were associated with salt tolerance in sugar beet^51^ and pea^13^. Here we report that the transcription levels of superoxide dismutase (*CuZn-SOD1*) and catalase (*CATb*) genes were significantly up-regulated in all salt-stressed rice and particularly highest in plants inoculated with strain GMKU 336. The results correlated with previous reports that PGPB reduced salt-induced lipid peroxidation through modulation of expression of ROS-scavenging enzymes^52^. The results indicated that strain GMKU 336 helps salt-stressed rice plants by reduction of lipid peroxidation and ROS levels and consequently promotes plant growth by induction of antioxidant enzymes.

In addition, up-regulation of *salT* was observed in all salt-stressed rice at the same expression level. *SalT* is one of the key genes for salt stress response that has been used as a marker for a salt-induced positive control gene^12^ whose transcript is not induced by ethylene^53^. We have shown up-regulation of *salT* in all salt stressed KDML105, supporting the view that this salt-sensitive cultivar changed in level of transcripts of salt stress responsive genes when exposed to salt.

**Figure 7:**
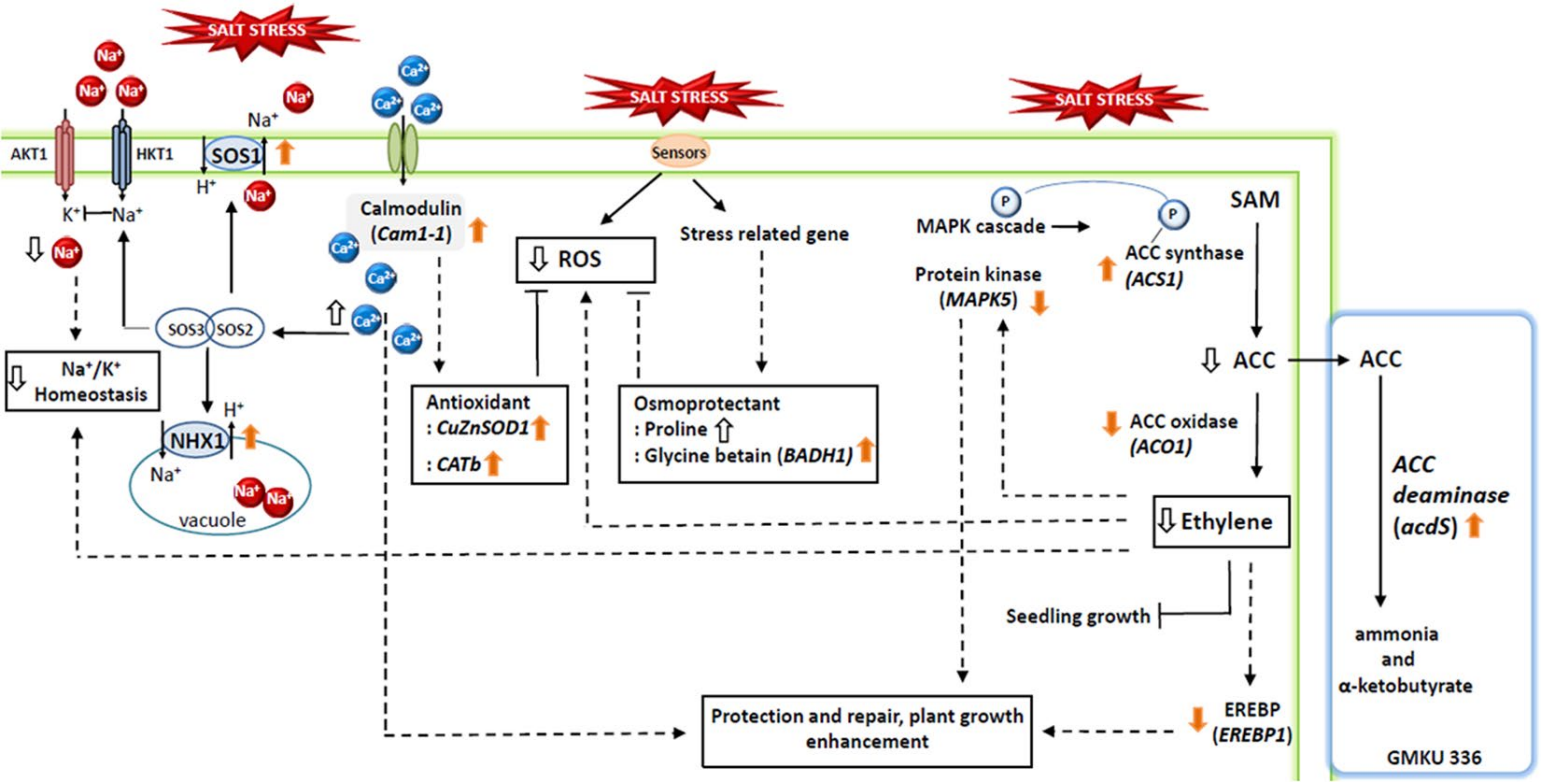
Molecular interaction scheme of ACCD-producing endophytic *Streptomyces* sp. GKU 336 associated with *Oryza sativa* L. cv. KDML105 under salt stress. Salt stress induces the ethylene biosynthesis pathway by up-regulation of *ACS1*. However, ACC is consumed by the *acdS* gene encoding ACCD of *Streptomyces* sp. GMKU 336, whereas *ACO1* and *EREBP1* are down-regulated and ethylene is reduced as a consequence. Depletion of ethylene induced less expression of *MAPK5* as well as lowering ROS accumulation. Salt tolerance in rice is enhanced by increases in proline and glycine betaine by up-regulation of the *BADH1* gene. Ca^2+^content is increased and Na^+^/K^+^ ratio is decreased which are correlated with up-regulation of *Cam1-1*, *SOS1* and *NHX1* genes. Ca^2+^ signal activates the SOS3/SOS2 protein kinase complex which negatively regulates the activity of Na^+^ ion channel. Association of Ca^2+^ and calmodulin activates antioxidant enzymes (*CuZn-SOD1* and *CATb*) which subsequently inhibits ROS. Bold orange arrow indicates gene regulation, bold white arrow indicates plant physiological regulation, black arrow indicates positive regulation, dashed arrow indicates indirect positive regulation, black line with bar end indicates inhibition, and dashed line with bar end indicates indirect inhibition.

In conclusion, all of the experimental data indicate that ACCD-producing endophytic *Streptomyces* sp. GMKU 336 promoted growth and protected salt-sensitive *Oryza sativa* L. cv. KDML105 from salt stress damage. This endophytic streptomycete enhanced salt tolerance in rice by lowering stress-induced ethylene via the action of ACCD; reduction of lipid peroxidation and Na^+^/K^+^ ratio but increasing Ca^2+^ content; chlorophyll content, accumulation of osmoprotectants: proline and glycine betaine. The plant physiology also correlated with expression profiles of stress responsive genes in rice associated with strain GMKU 336. The molecular interaction scheme of rice and *Streptomyces* sp. GMKU 336 under salt stress is summarized in Fig. 7. *AcdS* encoding ACCD of strain GMKU 336 was up-regulated *in vivo*, while *ACO1* and *EREBP1* were down-regulated and implicated in reduction of ethylene production in rice. Depleted ethylene induced less expression of *MAPK5*, that plays a role in salt tolerance as well as lowering ROS accumulation and consequently enhances plant growth. The presence of *Streptomyces* sp. GMKU 336 also enhances salt tolerance in rice by increasing proline and glycine betaine by up-regulation of *BADH1* gene expression to balance the osmotic pressure between rice tissues and salt-stress environment. Furthermore, an increase in Ca^2+^ and decrease in Na^+^/K^+^ homeostasis were correlated with up-regulation of the *Cam1-1* gene of calmodulin and *SOS1* and *NHX1* genes related to SOS pathway. The Ca^2+^ signal activates the SOS3/SOS2 protein kinase complex which negatively regulates the activity of the Na^+^ ion channel^13^. Consequently, the plasma membrane Na^+^/H^+^ antiporter (*SOS1*) was phosphorylated and drives the cytoplasmic Na^+^ into a vacuole, thus maintaining cellular ion homeostasis under salt stress. In addition, association of Ca^2+^ with calmodulin contributes to the antioxidant defense system by up-regulation of *CuZn-SOD1* and *CATb* gene expression to increase the production of antioxidant enzymes which subsequently inhibits ROS. Noticeably, plants inoculated with the ACCD-deficient mutant exhibited plant growth parameters, physiology and expression of all plant stress responsive genes in the same manner as those of un-inoculated controls. This supports the positive role of ACCD of *Streptomyces* sp. GMKU 336 in growth promotion and salt tolerance of rice.

It is clearly demonstrate for the first time that ACCD-producing *Streptomyces* sp. GMKU 336 enhances growth and salt tolerance by regulation of stress responsive genes of plants *in vivo* under salt-stress condition. Knowledge of the interaction is crucial to understand the relationship between rice plants and endophytic actinomycetes that is essential for further applications of endophytes as potential environmental friendly biofertilizers in saline soil.

## Methods

### Bacterial strain, identification and NaCl tolerance

*Streptomyces* sp. GMKU 336 was isolated from roots of a medicinal plant, *Clerodendrum serratum* (L.) Moon, collected from Khaohinson Royal Development Study Center, Chachoengsao Province, Thailand on starch casein agar (SCA)^9^. Its 16S rRNA gene was amplified and sequenced using primers listed in Supplementary Table S3^54,55^ (Genbank accession number KR870352). The sequence was analyzed and verified using EzTaxon-e database^56^. The NaCl tolerance of strain GMKU 336 was determined by growing on inorganic salt-starch agar (ISP-4) with addition of 1–12% (w/v) NaCl and incubated at 28 ± 2 °C for 14–21 days.

### Determination of phosphate solubilization, indole-3-acetic acid and siderophore production

*Streptomyces* sp. GMKU 336 was grown in tryptic soy broth (TSB) at 28 ± 2 °C, 200 rpm for 5 days. The cell culture was dropped onto Pikovskaya agar^57^ containing tricalcium phosphate and further incubated for 5 days. Presence of a clear zone indicates solubilization of phosphate.

Strain GMKU 336 was inoculated into TSB supplemented with tryptophan (500 μg mL^−1^) at 28 ± 2 °C, 200 rpm in the dark for 7 days. 1 mL supernatant was mixed with 2 mL Salkowski’s reagent^58^ and incubated for 30 min at room temperature. Development of a pink color indicates indole-3-acetic acid (IAA) production^58^.

A YM agar plug of 5-day growth of strain GMKU 336 was placed on a chrome azurol S (CAS) agar^59^ and incubated at 28 ± 2 °C for 3 days. An orange halo zone indicates siderophore production.

### Determination of ACC deaminase activity

*Streptomyces* sp. GMKU 336 was grown on mannitol soybean agar (MS) for 5 days and streaked on nitrogen-free minimal medium agar (MM), and MM agar supplemented with either 2 gL^−1^ (NH_4_)_2_SO_4_ or 3 mM ACC (Sigma-Aldrich) and incubated at 28 ± 2 °C in the dark for 7 days. Growth and sporulation on MM agar supplemented with ACC (MM-ACC) indicates ACC deaminase (ACCD) activity^4^.

For quantitative determination of ACCD activity, strain GMKU 336 was grown in TSB at 28 ± 2 °C, 200 rpm for 3 days. Cells were washed twice with 0.1 M Tris-HCl (pH 8.5) and resuspended in MM-ACC broth followed by incubation at 28 ± 2 °C, 200 rpm for 3 days. Cells were collected, washed twice and resuspended in 0.1 M Tris-HCl (pH 8.5). Cells were lysed by sonication and ACCD activity in the supernatant was assayed^7^. An aliquot of 200 mL of supernatant was incubated with 50 mM ACC at 30 °C for 1 h. The enzyme reaction was then stopped by adding 1.8 mL of 0.56 M HCl and 0.3 mL of 0.1% (w/v) 2,4-dinitrophenylhydrazine (prepared in 2 M HCl solution) and incubated at 30 °C for 15 min. The colorimetric reaction was then stopped by adding 2 mL of 2 M NaOH and the absorbance at 540 nm was determined by comparing to a standard curve of α-ketobutyrate.

### Construction of an ACCD-deficient mutant

Since *Streptomyces* sp. GMKU 336 was newly isolated and its genome has not yet been sequenced, partial ACCD gene (*acdS*) was obtained by PCR amplification using specific primers^9^ and annealing temperature listed in Supplementary Table S3. The primers were designed based on conserved amino acid regions^60^ with minimal degeneracy^9^. The PCR product was cloned into a non-replicative vector, pIJ8671^61^ to obtain pIJ8671/*acdS*. Next, pIJ8671/*acdS* was transformed into *E*. *coli* ET12567(pUZ8002)^62^ to perform an intergeneric conjugation^63^ with 24-h mycelium of strain GMKU 336. The mutants were selected by thiostrepton resistance (50 μg mL^−1^) and screened for deficiency of ACCD activity. Insertional inactivation of *acdS* in mutant was verified by PCR amplification using specific primers listed in Supplementary Table S3 of (i) thiostrepton resistance gene, (ii) partial *acdS* gene, and (iii) the absence of a 5.4-kb long PCR product (presence in pIJ8671/*acdS*) (Supplementary Fig. S1).

### Inoculation of rice plants with *Streptomyces* sp. GMKU 336 and its deficient mutant

Healthy seeds of Thai jasmine rice Khao Dok Mali 105 cultivar (KDML105), *Oryza sativa* L. cv. KDML105 were surface sterilized by immersion in 70% (v/v) ethanol for 1 min, 1% (w/v) sodium hypochlorite for 10 min and washed six times with sterile distilled water before transferring to a sterile moist chamber and incubation at room temperature in the dark for 3 days. Rice seedlings were grown under artificial light with light intensity at 8,000 lux for 16 h daily at room temperature for 7 days. The roots of seedlings was cut to the same length and were then immersed in individual sterile glass beakers containing 10^8^ spores mL^−1^ of *Streptomyces* sp. GMKU 336 or the mutant for 4 h. The seedlings were re-located to a moist sponge support for 1 day before transferring to a 20-L container filled with ½ Yoshida solution^64^ for 7 days and replaced with Yoshida solution for 7 days. Next, the nutrient solution was changed to Yoshida solution supplemented with 150 mM NaCl for 7 days. The pH of the nutrient solution was maintained between 5.0–5.5 throughout the growth period. A positive control of non-salt stressed rice was also grown under the same conditions without NaCl treatment.

Symptoms of salt toxicity were evaluated according to the standard evaluation system used at the International Rice Research Institute (IRRI)^10^.

Endophytic streptomycetes was re-isolated by surface-sterilized protocol as previously described^9^. The final washed solution was examined to ensure that the surface plant materials were actually sterilized. Re-isolated colonies of endophytic streptomycetes were randomly selected for analysis by 16S rDNA gene sequencing. In addition, re-isolated ACCD-deficient mutants were further verified by (i) growth on MS supplemented with thiostrepton (50 μg mL^−1^); (ii) deficiency of ACCD activity; and (iii) amplification of thiostrepton resistant gene.

### Determination of chlorophyll and ethylene contents

Approximately 100 mg of leaf fresh weight was ground in liquid nitrogen and chlorophyll was extracted twice by adding 1.0 mL DMSO and then sonicated^65^. The chlorophyll content of the supernatants were measured at 645 and 663 nm within 20 min after the extraction.

Ethylene production was measured by enclosing the whole rice plants in a 250-mL sealed glass container containing 50 mL acetylene for 1 h. A 1 mL gas sample was withdrawn and quantified by gas chromatography at PGPR Biofertlizer and Aerated Compost Soil Microbiology Research Group, Soil Science Division, Department of Agriculture, Ministry of Agriculture and Cooperatives, Bangkok, Thailand.

### Determination of proline, ion, and relative water contents

250 mg fresh weight of the whole plantlets were ground in liquid nitrogen and mixed with 5 mL aqueous sulfosalicylic acid (3% w/v) and filtered through Whatman^®^ No. 1. 1 mL of filtrate was mixed with an equal volume of glacial acetic acid and ninhydrin reagent (1.25 mg ninhydrin, 30 mL glacial acetic acid, and 20 mL 6 M H_3_PO_4_) and incubated for 1 h at 100 °C in boiling water. The reaction was terminated by placing the test tube in an ice bath. Next, the reaction mixture was vigorously mixed with 2 mL toluene. After warming at 25 °C, proline was measured at 520 nm^66^.

Rice shoots were assayed for Na^+^, K^+^ and Ca^2+^ contents at the Department of Soil Science, Faculty Agriculture, Kasetsart University, Bangkok, Thailand.

The relative water content (RWC) of plant leaves was examined^67^. ~10 cm of leaf was cut off from the middle part of the youngest fully expanded leaf, weighed and placed in a tube. The tube was kept on ice and was filled with distilled water and kept in the dark at 4 °C overnight. The leaf was blotted dry and weighed. The samples were then dried at 70 °C for 3 days and weighed. The RWC was calculated from each weigh^66^.

### ROS staining and estimation of lipid peroxidation

For detection of superoxide^68^, rice leaves were immersed in 25 mL of nitrobluetrazolium (NBT) solution (0.5 μg mL^−1^ NBT in 10 mM phosphate buffer, pH 7.6) for 3 h in the dark. For detection of hydrogen peroxide^69^, leaves were immersed in 25 mL 3,3′-diaminobenzidine (DAB) solution (1 μg mL^−1^ DAB in 50 mM Tris-acetate buffer, pH 5.0) for 8 h. After staining, both treatments were boiled in 95% (v/v) ethanol for 30 min. The leaves were then immersed in 40% glycerol for 16 h before color detection.

The amount of lipid peroxidation was determined by estimating malondialdehyde (MDA)^69^. Rice shoots were ground in 80% (v/v) ethanol (1 g fresh weight 25 mL^−1^). 1 mL aliquots of samples were added with 1 mL of either (i) −TBA solution [20% (w/v) trichloroacetic acid and 0.01% butylated hydroxytoluene], or (ii) +TBA solution containing –TBA solution plus 0.65% thiobarbituricacid (TBA) and mixed vigorously before heating at 95 °C for 25 min. Absorbance was determined at 440 nm, 532 nm, and 600 nm and MDA equivalents were calculated^69^.

### Streptomycete RNA extraction and semi qRT-PCR analysis

*Streptomyces* sp. GMKU 336 was grown in TSB at 28 ± 2 °C, 200 rpm for 3 days. Cells were washed twice with 0.1 M Tris-HCl (pH 8.5) and resuspended in MM-ACC broth followed by incubation at 28 ± 2 °C, 200 rpm for 3 days. Total RNA was extracted following the manufacturer’s protocol of Trizol^®^ Reagent (Invitrogen). cDNA was synthesized using the Thermo Scientific RevertAid First strand cDNA synthesis Kit (Thermo Scientific). Semi-quantitative RT-PCR analysis was performed using cDNA products, the corresponding primers listed in Supplementary Table S3, and Phusion^®^ Hot Start II-High Fidelity DNA polymerase (Thermo Scientific). The expression level of each product was quantified by Gel Doc^TM^ XR + with Image Lab^TM^ Software (Biorad) and normalized against the expression of a housekeeping gene, *hrdB*^70^.

### Rice RNA extraction and transcription analysis of genes involved in salt stress response by real-time PCR

Total RNA was extracted from shoots following the manufacturer’s protocol for Trizol^®^ Reagent (Invitrogen) and treated with DNase I (Thermo Scientific). cDNA was synthesized using the Thermo Scientific RevertAid First strand cDNA synthesis Kit (Thermo Scientific). KAPA SYBR^®^ FAST qPCR Master Mix (2x) (KAPA BIOSYSTEMS) was used for quantification in Master Cycler Realplex 4 (Eppendorf). The primers for real-time PCR are listed in Supplementary Table 3^71–74^. The mean value was calculated and normalized with actin (*act1*) as internal control.

### Statistical analysis

All data from the experiments were calculated and statistically evaluated from biological and technical triplicates. The data were analyzed with one way analysis of variance (ANOVA) and Duncan’s test to determine any significant differences between groups at P < 0.05. All statistical analyses were performed using the SPSS 20.0 for Windows software (SPSS Inc).

## Electronic supplementary material

Supplementary Figures and Tables
