# Supplementary material for: Molecular interaction of 1-aminocyclopropane-1-carboxylate deaminase (ACCD)-producing endophytic Streptomyces sp. GMKU 336 towards salt-stress resistance of Oryza sativa L. cv. KDML105
Source: Sci Rep. 2018 Jan 31;8:1950. doi: 10.1038/s41598-018-19799-9 (PMC5792428; doi:10.1038/s41598-018-19799-9)
Supplement: Supplementary file 1 — Supplementary Figures and Tables [file 41598_2018_19799_MOESM1_ESM.pdf]

Supplementary Figures and Tables

Molecular interaction of 1-aminocyclopropane-1-carboxylate deaminase (ACCD)-producing endophytic *Streptomyces* sp. GMKU 336 towards salt-stress resistance of *Oryza sativa* L. cv. KDML105

Ratchaniwan Jaemsaeng[1,2], Chatchawan Jantasuriyarat[1] and Arinthip Thamchaipenet [1,2*]

[1]Department of Genetics, Faculty of Science, Kasetsart University, Bangkok 10900, Thailand

[2]Center for Advanced Studies in Tropical Natural Resources, National Research University-KasetsartUniversity (CASTNAR, NRU-KU), Bangkok 10900, Thailand

*Correspondence and requests for materials should be addressed to A.T. (e-mail: arinthip.t@ku.ac.th)

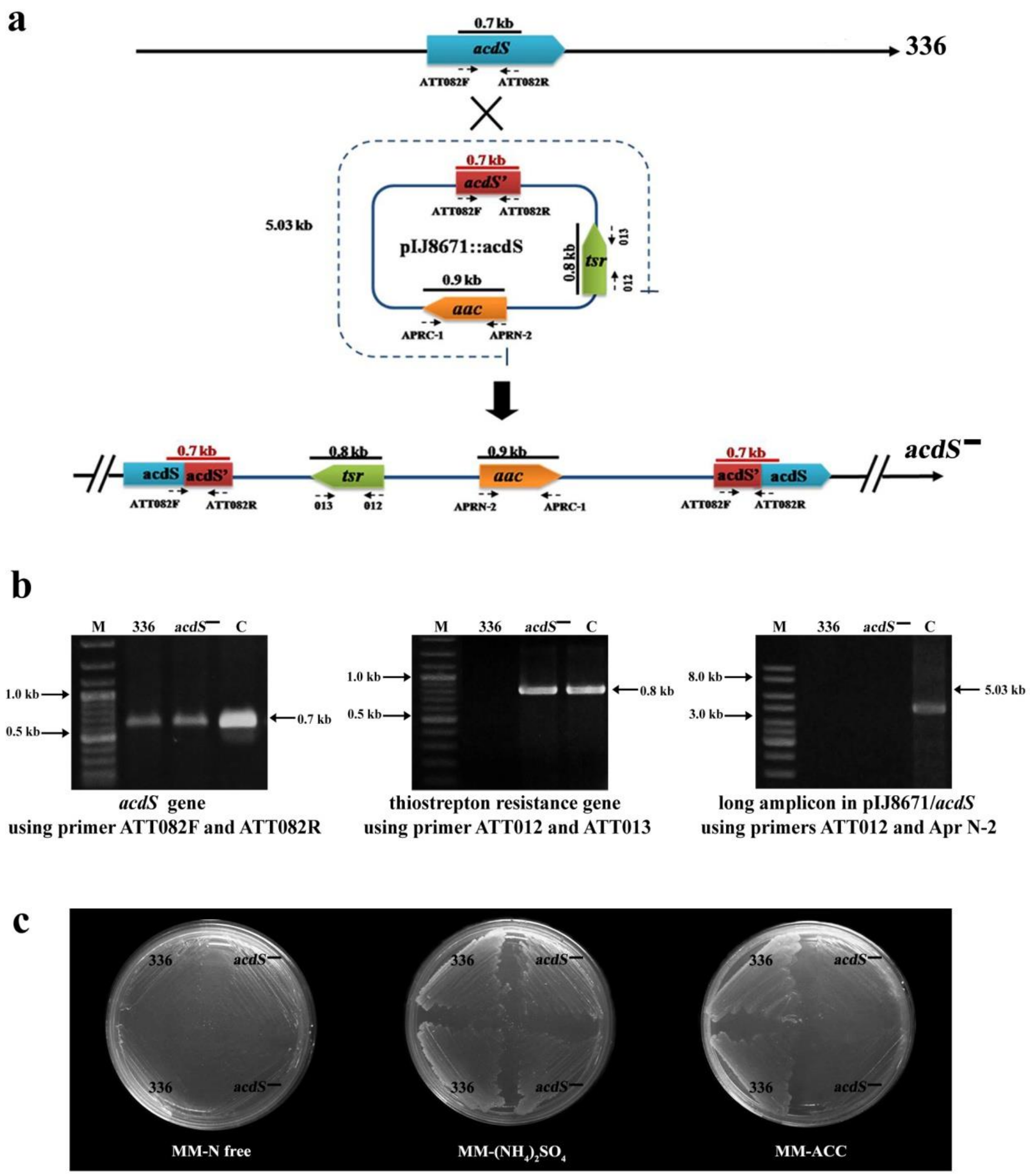

Supplementary Figure S1. Characterization of *Streptomyces* sp. GMKU 336 and the ACCD-deficient mutant. (a) Illustration of the insertion inactivation of the *acdS* gene. Small dashed arrows indicate primers used for PCR amplification tests; (b) PCR amplification tests for *acdS* gene, thiostrepton resistant gene and long amplicon in pIJ8671/*acdS*; (c) ACC deaminase activity on MM-N free, MM-$(NH_4)_2SO_4$, and MM-ACC media. M, 1-kb ladder; C, pIJ8671/*acdS*; 336, *Streptomyces* sp. GMKU 336; *acdS*⁻, ACCD-deficient mutant.

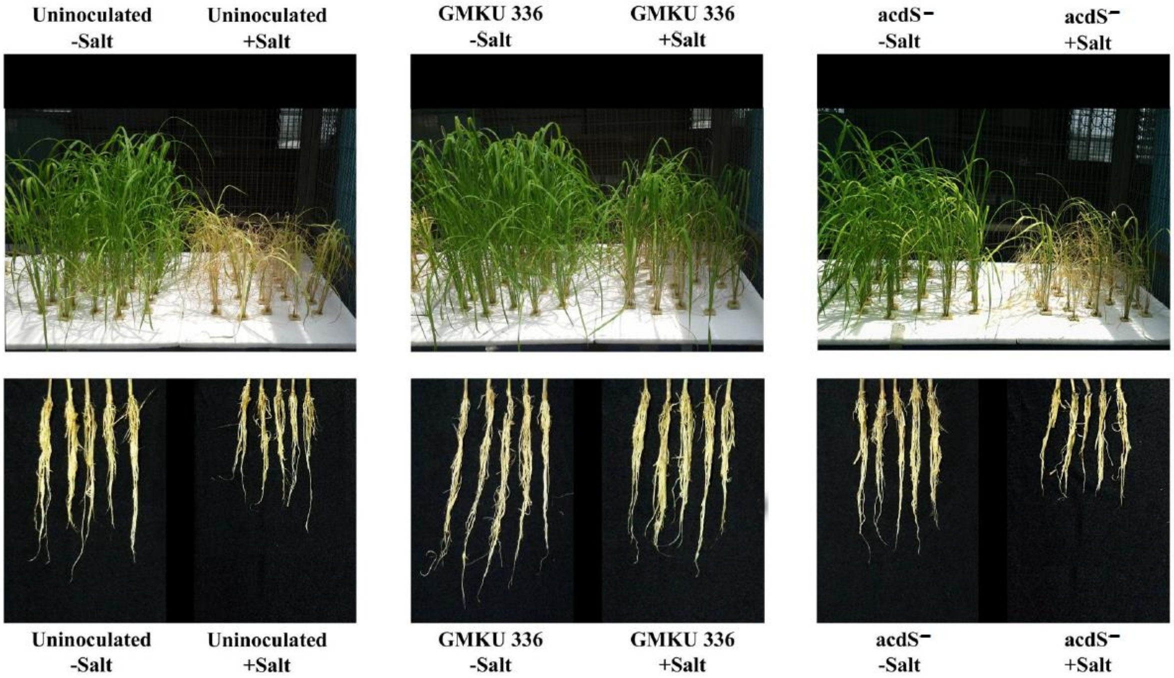

Supplementary Figure S2. Effect of ACCD-producing endophytic *Streptomyces* sp. GMKU 336 on *Oryza sativa* L. cv. KDML105 after 7 days of salt stress under hydroponic conditions. Uninoculated, plants without bacteria inoculation; GMKU 336, plants inoculated with *Streptomyces* sp. GMKU 336; acdS^{-}, plants inoculated with ACCD-deficient mutant; -Salt, non-salt treatment; +Salt, salt treatment (150 mM NaCl).

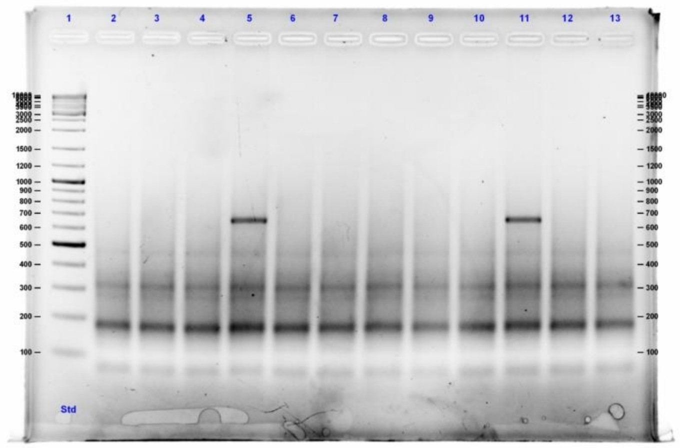

(a)

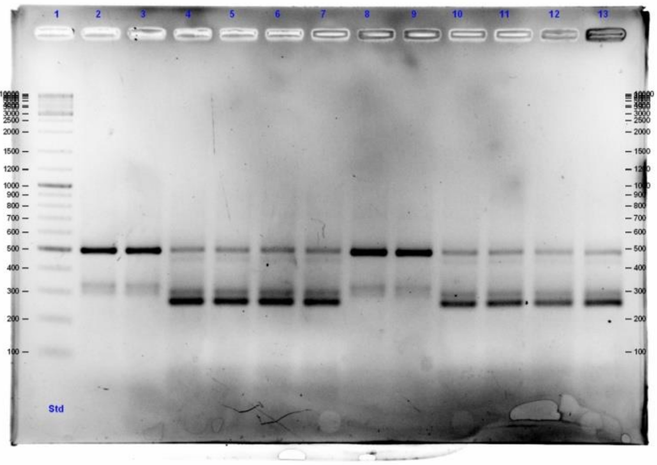

(b)

Supplementary Figure S3. Transcriptional analysis by qRT-PCR of *acdS* and *hrdB* genes of *Streptomyces* sp. GMKU 336 associated with KDML105 in vivo. (a) *acdS* gene (700 bp); (b) *hrdB* gene (250 bp); Lane 1, 1 kb ladder; Lane 2, un-inoculated control plants; Lane 3, un-inoculated salt-stress plants; Lane 4, *Streptomyces* sp. GMKU 336 inoculated plants; Lane 5, *Streptomyces* sp. GMKU 336 inoculated salt-stress plants; Lane 6, ACCD-deficient mutant inoculated plants; Lane 7, ACCD-deficient mutant inoculated salt-stress plants; Lane 8, 9, 10, 11, 12, and 13 are replicates of Lane 2, 3, 4, 5, 6, and 7, respectively.

Supplementary Table S1 Plant growth and physiological parameters of *Oryza sativa* L. cv. KDML105 with and without ACCD-producing *Streptomyces* sp. GMKU 336 and ACCD-deficient mutant under salt (150 mM NaCl) and non-salt treatments.

**Parameters**	**Treatments**					
	Uninoculated -Salt	Uninoculated +Salt	GMKU 336 -Salt	GMKU 336 +Salt	acdS^{-} -Salt	acdS^{-} +Salt
Shoot length (cm)	78.70±1.61^{ab}	60.40±1.05^{d}	81.8±1.53^{a}	70.60±1.87^{c}	76.40±1.28^{b}	58.23±0.91^{d}
Root length (cm)	20.87±0.49^{ab}	15.60±1.06^{c}	23.17±0.90^{a}	20.43±0.78^{b}	19.00±0.82^{ab}	16.20±1.05^{c}
Shoot fresh weight (g)	0.90±0.01^{b}	0.54±0.05^{d}	1.50±0.03^{a}	0.66±0.031^{c}	0.89±0.08^{b}	0.52±0.05^{d}
Root fresh weight (g)	0.19±0.01^{b}	0.12±0.01^{d}	0.27±0.03^{a}	0.16±0.01^{c}	0.19±0.01^{b}	0.16±0.01^{d}
Shoot dry weight (g)	0.18±0.01^{b}	0.08±0.01^{d}	0.28±0.01^{a}	0.12±0.01^{c}	0.16±0.01^{b}	0.07±0.02^{d}
Root dry weight (g)	0.05±0.01^{bc}	0.02±0.01^{d}	0.06±0.01^{a}	0.03±0.01^{cd}	0.05±0.01^{ab}	0.02±0.01^{d}
Total chlorophyll (mg g FW^{-1})	0.66±0.02^{a}	0.27±0.01^{c}	0.66±0.02^{a}	0.46±0.01^{b}	0.65±0.01^{a}	0.30±0.01^{c}
Ethylene production (µmol g DW^{-1} h^{-1})	0.98±0.13^{b}	2.15±0.11^{a}	0.78±0.06^{b}	1.01±0.10^{b}	0.97±0.13^{b}	1.93±0.06^{a}
RWC (%)	98.44±0.44^{a}	80.56±0.97^{c}	98.54±0.47^{a}	91.91±0.32^{b}	98.23±0.53^{a}	81.26±1.16^{c}
Proline content (µg g FW^{-1})	8.13±0.76^{c}	23.83±0.18^{b}	8.73±1.15^{c}	28.97±0.48^{a}	8.63±0.41^{c}	24.80±1.73^{b}
Na^{+} content (mg g DW^{-1})	3.33±0.68^{c}	30.60±3.40^{a}	3.10±0.46^{c}	18.63±1.12^{b}	2.77±0.31^{c}	27.83±2.14^{a}
K^{+} content (mg g DW^{-1})	44.90±3.97^{a}	31.93±1.63^{c}	44.83±4.68^{a}	38.37±2.75^{b}	45.27±2.32^{a}	29.27±1.60^{c}
Na^{+}/K^{+}	0.08±0.02^{c}	0.96 ±0.07^{a}	0.07±0.02^{c}	0.49±0.04^{b}	0.06±0.01^{c}	0.95±0.09^{a}
Ca^{2+} content (mg g DW^{-1})	8.10±0.20^{a}	6.13±0.40^{c}	7.90±0.46^{a}	6.90±0.30^{b}	8.10±2.32^{a}	6.23±0.31^{c}
MDA content (uM g FW^{-1})	29.40±0.10^{c}	55.63±1.19^{a}	28.93±0.72^{c}	33.07±1.72^{b}	28.93±1.05^{c}	55.67±0.64^{a}
Salt injury score	ND	7.40±0.40^{a}	ND	3.20±0.36^{b}	ND	7.60±0.52^{a}
Salt tolerant type	ND	Susceptible	ND	Tolerant	ND	Susceptible
Colonization (10^{4} CFU g root FW^{-1})	0.00	0.00	8.50±1.40	4.66 ±3.05	5.48 ±3.47	4.84 ±3.19

Values are mean of three replicates ± standard error of mean. Different letters indicated statistical differences between treatments (Duncan's test, P<0.05). Uninoculated, plants without bacteria inoculation; GMKU 336,

plants inoculated with *Streptomyces* sp. GMKU 336; acdS⁻, plants inoculated with ACCD-deficient mutant; -Salt, non-salt treatment; +Salt, salt treatment (150 mM NaCl); DW, dry weight; FW, fresh weight; ND, not determined.

SupplementaryTable S2 Transcriptional levels of genes involved in salt stress response in *Oryza sativa* L. cv. KDML105 with and without ACCD-producing *Streptomyces* sp. GMKU 336 and ACCD-deficient mutant under salt (150 mM NaCl) and non-salt treatments.

**Relative expression**	**Treatments**					
	Uninoculated -Salt	Uninoculated +Salt	GMKU 336 -Salt	GMKU 336 +Salt	acdS⁻ -Salt	acdS⁻ +Salt
ACS1	0.96±0.20^{b}	4.56±0.66^{a}	0.48±0.21^{b}	4.72±0.87^{a}	0.90±0.27^{b}	5.21±0.08^{a}
ACO1	0.78±0.24^{c}	2.46±0.35^{a}	0.41±0.17^{c}	1.51±0.07^{b}	0.29±0.16^{c}	3.02±0.41^{a}
EREBP1	0.74±0.23^{c}	2.60±0.17^{a}	0.49±0.31^{c}	1.69±0.21^{b}	0.31±0.02^{c}	2.86±0.33^{a}
salT	0.98±0.09^{b}	1.27±0.06^{a}	0.93±0.11^{b}	1.44±0.30^{a}	0.70±0.16^{b}	1.37±0.05^{a}
BADH1	0.87±0.14^{c}	1.65±0.04^{b}	0.90±0.10^{c}	4.42±0.32^{a}	0.93±0.15^{c}	1.68±0.15^{b}
NHX1	0.87±0.13^{c}	1.42±0.14^{b}	1.04±0.09^{c}	2.53±0.30^{a}	0.88±0.10^{c}	1.60±0.10^{b}
SOS1	0.87±0.13^{c}	2.02±0.42^{b}	0.65±0.07^{c}	3.09±0.13^{a}	0.70±0.10^{c}	0.13±0.13^{b}
Cam1-1	0.95±0.11^{c}	1.69±0.15^{b}	1.10±0.11^{c}	3.23±0.57^{a}	0.84±0.08^{c}	1.75±0.07^{b}
MAPK5	0.95±0.18^{c}	3.23±0.17^{a}	0.85±0.14^{c}	1.83±0.12^{b}	0.91±0.11^{c}	3.21±0.21^{a}
CuZn-SOD1	0.99±0.04^{c}	2.27±0.12^{b}	0.73±0.24^{c}	3.67±0.31^{a}	0.97±0.06^{c}	2.34±0.13^{b}
CATb	0.93±0.08^{c}	2.10±0.10^{b}	0.97±0.09^{c}	3.00±0.05^{a}	0.94±0.37^{c}	1.99±0.21^{b}

Values are mean of three replicates ± standard error of mean. Different letters indicated statistical differences between treatments (Duncan's test, $P<0.05$). Uninoculated, plants without bacteria inoculation; GMKU 336, plants inoculated with *Streptomyces* sp. GMKU 336; acdS⁻, plants inoculated with ACCD-deficient mutant; -Salt, non-salt treatment; +Salt, salt treatment (150 mM NaCl).

Supplementary Table S3 List of primers used in this study

Primers	Sequence (5'–3')	Description (gene)	T_a (°C)	Reference
926F	AAACTCAAAGGAATTGACGG	16S rDNA for sequencing		54
ATT026	TGGACTACCAGGGTATCTAATC			
1530R	AAGGAGATCCAGCCGCA	16S rDNA	55	55
STR1F	TCACGGAGAGTTTGATCCTG			
aco-F	GATAGCGTGTGTACCACAGCGACC	*ACO1*	64	71
aco-R	CACGGTACAGCACGCCGCAC			
acs-F	GCCGAATTCGATGGTGAGCCAAGTGGTCG	*ACS1*	57	72
acs-R	GAGCGCGTGGGGGTTCTTCT			
act-F	AGCTATCGTCCACAGGAA	*act1*	49	73
act-R	ACCGGAGCTAATCAGAGT			
Apr N-2	CCCCGGCGGTGTGCT	5.4 kb long amplicon	69	3
ATT012	TGACTGAGTTGGACACCATCGC			
ATT012	TGACTGAGTTGGACACCATCGC	*thio*^{r}	58	63
ATT013	CCTGTCGATCCTCTCGTGCAG			
ATT082F	CAGTCCAACCACACSCGSCAG	*acdS*	65	This work
ATT082R	GCCATSGACTTSCCCTCGTASAC			
BADH-For	TAGCTTCACATCCCCATGTG	*BADH1*	57	12
BADH_R	TAGACGAGAAGTAGCACTGC			This work
CATb_FW	ATGGATCCCTACAAGCATCG	*CATb*	57	12
CATb_R	GGCTCCCCTTGCATGAACGAC			This work
CuZn-SOD1F	TTAACAATGGTGAAGGCTG	*CuZn- SOD*	57	This work
CuZn-SOD1R	TGTAGTGTGGCCCAGTTGA			
EREBP-For	GGGCCCTCTCCAGAGATAAC	*EREBP1*	57	12
EREBP-Rev	TGCAGCTTCTTCAGCACTGT			
hrdBF	CCGAGTCTGTGATGGCGCTC	*hrdB*	62	70
hrdBR	TTGGTGGCGGTGCGCTTGAC			
MAPK-For	CGACATGATGACGGAGTACG	*MAPK5*	57	12
MAPK5_R	CGTCCTCGTTCCGTATG			This work
NHX-For	GCTAGATTTGAGCGGCATTC	*NHX1*	57	12
NHX1-R	TCAATATCCAATGCATCCATC			This work
OsCam1-1F	ACCGTGCATTGCCGTATTAG	*Cam1-1*	57	74
OsCam1-1R	GCAAGCCTTAACAGATTCAC			
salT-F	GGAATATGCCATTGGTCCAT	*salT*	55	11
salT-R	GTCTTGCAGTGGAATGCTGA			
SOS-For	TCTAGTCGTTGCCAGGCTTT	*SOS1*	55	12
SOS-Rev	TCATTGATCATGCTCCCGTA			This work
